# Detrimental Effects of Alcohol on the Heart: Hypertension and Cardiomyopathy

**DOI:** 10.31083/j.rcm2410292

**Published:** 2023-10-17

**Authors:** Vincent M. Figueredo, Akash Patel

**Affiliations:** ^1^Department of Cardiology, St Mary Medical Center, Langhorne, PA 19047, USA

**Keywords:** alcoholic, alcohol abuse, cardiomyopathy, hypertension, heart failure

## Abstract

**Background:**

Epidemiological evidence suggests a J-shaped association between alcohol consumption and cardiovascular mortality, with higher cardiovascular event rates occurring among abstainers and heavy drinkers compared to moderate consumers. However, this hypothesis has been challenged by more recent studies. Furthermore, ethnicity, gender, type of alcoholic beverage, and pattern of alcohol intake, influence the relationship between alcohol and heart health.

**Methods:**

We undertook a review of the relavent literature utilizing PubMed.

**Results:**

Heavy alcohol consumption causes resistant hypertension, cardiomyopathy, arrhythmias, hemorrhagic strokes, as well as hepatic cirrhosis and pancreatitis. Excessive drinking is the third most preventable cause of death worldwide behind hypertension and smoking.

**Conclusions:**

In this review, we discuss the effects of alcohol abuse on hypertension (a major cause of myocardial infarction and stroke) and alcoholic cardiomyopathy. Another article in this Special Issue “Alcohol and Heart Health” discusses the problem with alcohol and arrhythmias sudden cardiac death.

## 1. Introduction

Alcohol, a psychoactive substance, has been a significant part of human culture for millennia. Throughout history it has been recognized for its harmful potential to individuals, as well as to society, due to its dependence-producing properties. Harmful alcohol consumption contributes to health, social, and economic burdens globally.

Significant alcohol consumption remains highly prevalent globally. In 2018, alcohol consumption averaged 6.4 liters of pure alcohol per person worldwide [1]. In the United States, 85% of individuals 18 years-old or older have consumed alcohol in their lifetime [2]. Furthermore, binge drinking, a major reason for alcohol related problems, is widespread. According to a United States governmental survey in 2019, 26% of individuals aged 18 and older reported binge drinking habits within the last month [3].

The abusive use of alcohol costs billions of dollars globally. In 2010, excessive alcohol consumption cost the healthcare sector approximately 28 billion dollars [4]. If damages related to workplace productivity, crime, and accidents were included, the costs is more than 250 billion dollars a year [4]. The World Health Organization (WHO) reported that excessive alcohol use is a risk factor for more than 200 diseases, accounting for more than 5% of the global burden of diseases and injuries [1]. Furthermore, it acts as a common risk factor for mental disorders and abnormal behavioral conditions [1]. The Organization for Economic Cooperation and Development Strategic Public Health Planning for Non-Communicable Diseases (OECD SPHeP-NCDs) model predicts that consuming more than 1 drink per day for women and 1.5 drinks per day for men will cause 24 million new cases of cardiovascular diseases, 10 million cancer cases,12 million new diabetes cases, and 37 million injury cases between 2020–2050 in 52 countries [5]. Consistent excessive drinking can lead to alcohol use disorder (AUD). Individuals with this condition consume too much alcohol despite the mental and physical harms. In USA, approximately 15 million individuals had been diagnosed with AUD [6].

Excessive alcohol consumption causes 3 million deaths yearly, and it accounts for 5% of all deaths worldwide. It was the 7th leading cause of early death globally in 2016 [7]. In addition, 14% of all deaths of individuals between age 20 and 39 in 2016 were alcohol consumption related [1]. Most alcohol attributable deaths (28.7%) in 2016 were due to injuries. Other complications were cardiovascular diseases (19%), liver cirrhosis and pancreatitis (21.3%), and cancer (12.6%) [1]. Due to increased alcohol consumption seen in adults worldwide, an increased focus needs to be placed on the cardiovascular harms of heavy drinking.

Heavy alcohol use is defined as greater than 4 drink per day or 14 drinks per week for men and greater than 3 drinks per day or 7 drinks per week for women [8]. While numerous studies suggest a cardioprotective effect of light to moderate alcohol consumption, especially on coronary artery disease, heavy alcohol consumption, abuse and binging, can negatively affect the cardiovascular system. Potential cardiovascular detrimental effects include congestive heart failure, dilated cardiomyopathy, sudden cardiac death and hemorrhagic stroke. Some of these cardiovascular complications are the result of alcohol-induced hypertension. In this review, we discuss the effects of alcohol abuse on hypertension (a major cause of myocardial infarction and stroke) and alcoholic cardiomyopathy. Another article in this Special Issue “Alcohol and Heart Health” discusses the problem with alcohol and arrhythmias and sudden cardiac death.

## 2. Alcohol and Hypertension

Hypertension is the most common medical condition in the world, with 1.28 billion cases in 2021 [9]. Hypertension is a major cause of premature death worldwide [10]. Moreover, many individuals with hypertension are unaware that they have it (~46%) [9]. It is a risk factor for stroke, cardiovascular and kidney diseases [9, 10]. According to WHO, increased alcohol consumption is a major risk factor for developing hypertension [9]. The correlation between alcohol consumption and hypertension had been investigated in cross sectional, cohort and epidemiological studies across various populations. Meta-analyses of these studies have also been performed and published in the last decade which provide the strongest evidence for an association between alcohol and hypertension.

Roerecke *et al*. [11] conducted a meta-analysis investigating the incidence of hypertension in moderate drinking individuals. Their analysis included 361,254 participants from 20 original cohort studies. They found the relationship between alcohol and hypertension is dependent on gender and dose. Any amount of drinking was shown to be associated with more hypertension in men. In women, no risk was found at 1 to 2 drinks per day, but greater risk was found at higher levels of consumption [11]. Similar results were observed by Aladin and colleagues [12]. They found that moderate drinkers (7–13 drinks/week) were at increased risk for stage 1 and stage 2 hypertension, when compared to never drinkers, in 17,059 subjects from NHANES III (Third National Health and Nutrition Examination Survey) [12].

Another meta-analysis by Roerecke *et al*. [13], investigating whether reducing the intake of alcohol had any effect on blood pressure, studied data from 36 trials. They demonstrated that individuals who had ≤2 drinks per day had no effect on their blood pressure when alcohol consumption was reduced, while individuals consuming >2 drinks per day had significant reductions in their blood pressure with reduced alcohol intake [13].

McFadden and colleagues [14], studying the effects of daily alcohol consumption on blood pressure, found that there is a statistically significant increase in blood pressure immediately after alcohol intake.

Chen *et al*. [15] applied a Mendelian randomization approach to study the effects of alcohol on blood pressure in subjects with the alcohol dehydrogenase 2 (ALDH2) genotype (aldehyde dehydrogenase 2 is a major enzyme in alcohol metabolism). They concluded that alcohol intake had a significant immediate effect on blood pressure, as well as it increasing the risk for hypertension [15].

Jung *et al*. [16] recently conducted a regional analysis of the relationship between alcohol and hypertension. They found that the risk of hypertension is dose dependent in both Asian and western populations [16]. Further, hypertension risk is evident even at lower levels of consumption compared to recommended guidelines. Asian populations showed a higher risk of hypertension at low doses of alcohol compared to a western population [16]. Another meta-analysis showed that even at low to moderate doses of alcohol, men had an increased trend towards development of hypertension, when compared to women [17]. Table 1 (Ref. [18, 19, 20, 21, 22, 23, 24, 25, 26, 27, 28, 29]) shows study characteristics for studies included in the above reviews and meta-analyses examining alcohol use and hypertension.

**Table 1.** **Study characteristics comparing alcohol use and hypertension**.

Study	Study design	Country	Number of participants	% Men and women	Average age (years)	Alcohol consumption	HR/OR/RR (95% Confidence Interval)
Bae* et al*., 2002 [18]	Nested Case Control	South Korea	988	100% Men	40–59	71–280 g/week	OR:
							1.84 (1.31–2.56)
Sesso *et al*., 2008 [19]	Prospective Cohort	United States of America	42,303	32% Men	Men: 52	5–6 drinks/week	RR:
				68% Women	Women: 53.8		Men: 1.15 (1.04–1.27)
							Women: 0.90 (0.80–1.00)
Forman *et al*., 2009 [20]	Prospective Cohort	United States of America	83,882	100% Women	27–44	5.1–10.0 g/day	HR:
						10.1–15.0 g/day	0.84 (0.78–0.90)
						15.1–29.9 g/day	0.98 (0.91–1.07)
						≥30 g/day	1.11 (1.01–1.23)
							1.61 (1.42–1.82)
Ascherio *et al*., 1996 [21]	Prospective Cohort	United States of America	41,541	100% Women	38–63	20–29.9 g/day	RR:
						≥30 g/day	1.46 (1.21–1.76)
							1.32 (1.11–1.57)
Halanych *et al*., 2010 [22]	Prospective Cohort	United States of America	659	56.6% Men	18–30	7–14+ drinks/week	HR:
				43.4% Women			1.33 (0.76–2.32) African-American Men
							1.47 (0.44–4.96) European-American Men
Peng *et al*., 2013 [23]	Prospective Cohort	China	32,389	74.2% Men	49.9	50–99 g/day	RR:
				25.8% Women		100–149 g/day	1.80 (1.63–2.00)
						≥150 g/day	2.06 (1.83–2.31)
							2.28 (1.99–2.61)
Okubo *et al*., 2014 [24]	Prospective Cohort	Japan	45,428	35.6% Men	40–79	20–39.9 g/day	HR:
				64.4% Women		20–59.9 g/day	Men:
						≥60 g/day	1.29 (1.24–1.35)
							1.45 (1.39–1.52)
							1.57 (1.46–1.70)
							Women:
							1.10 (1.03–1.17)
							1.14 (0.93–1.40)
							1.29 (0.89–1.87)
Diederichs *et al*., 2017 [25]	Prospective Cohort	Germany	2231	47.5% Men	18–79	≥10–20 g/day	OR:
				52.5% Women			Men: 2.88, *p*-value 0.014
							Women: 2.13, *p*-value 0.092
Saremi *et al*., 2004 [26]	Prospective Cross Sectional	United States of America	3789	34.2% Men	36.3	1–2 drinks/day	HR:
				65.8% Women			Men: 1.20 (0.95–1.53)
							Women: 1.48 (1.24–1.78)
Yoo *et al*., 2019 [27]	Prospective Cohort	South Korea	6259	34.5% Men	40–69	≥5 and <30 g/day	HR:
				65.5% Women			Men: 1.292 (1.033–1.617)
							Women: 1.128 (0.652–1.952)
Fuchs *et al*., 2001 [28]	Prospective Cohort	United States of America	8334	33.2% White Men	45–64 years	1 to 209 g/week	OR:
				40% White Women			White men: 0.88 (0.71–1.08)
				10.0% Black Men			White women: 0.89 (0.73–1.09)
				16.8% Black Women			Black men: 1.71 (1.11–2.64)
							Black women: 0.88 (0.59–1.33)
Ohmori *et al*., 2002 [29]	Prospective Cohort	Japan	267	39% Men	40+	23–45 g/week	RR:
				61% Women			Men: 2.60 (1.50–4.49)

Table notes: OR, odds ratio; HR, hazard ratio; RR, relative risk.

Thus, the risk of hypertension is dose dependent with higher use of alcohol contributing to higher hypertension risk. Other factors which contribute include gender, timing of alcohol consumption (e.g., binging), and genetics.

## 3. Dilated Cardiomyopathy

Alcoholic cardiomyopathy is a type of dilated cardiomyopathy (DCM) demonstrating increased left ventricular (LV) mass and decreased ventricular function. Alcoholic cardiomyopathy is similar to other dilated non-ischemic cardiomyopathies. The majority of alcohol abusers are asymptomatic for years. Most never develop clinical manifestations of congestive heart failure. However, most alcoholics do demonstrate preclinical heart muscle disease. Autopsies reveal dilated cardiomyopathic in alcoholics not experiencing symptomatic heart failure [30]. It has been estimated that 2% of heavy alcohol users ultimately develop symptomatic alcoholic cardiomyopathy. And because there are so many alcoholics, approximately 35% of all non-ischemic cardiomyopathies are caused by excessive alcohol use [31, 32, 33].

Data suggest that most alcoholics develop significant changes in cardiac function and myocyte structure after consuming on average greater than 90 grams of alcohol daily for at greater than 5 years [34, 35, 36, 37]. Cardiac damage due to longstanding heavy alcohol consumption is not beverage or quantity specific and varies depending on the population studied. Genetic and environmental factors play a role, as well as the specific beverage type used by a culture or individual.

Studies have examined the association between alcohol abuse and cardiomyopathy [31, 32, 33]. In one case control study researchers found that 40% of DCM cases were attributable to excessive alcohol use history [38]. In another case control study by Komajda *et al*. [39], abnormal use of alcohol was found to be strong predictor for cardiomyopathy cases irrespective of the type of beverage used. Gillet *et al*. [40] found that the higher use of alcohol (>82 g/day) was seen in DCM group compared to a control group (32 g/day) in a French population. Whitman and colleagues [41] performed a prospective cohort study on 268,084 alcoholics in California, United States, using the Healthcare Cost and Utilization Project database. They found a significant increase in congestive heart failure cases compared to the non-alcoholic population, with a hazard ratio of 2.34. Table 2 (Ref. [41, 42, 43, 44, 45, 46, 47, 48]) shows study characteristics for studies included in the above reviews and meta-analyses examining alcohol use and congestive heart failure.

**Table 2.** **Study Characteristics Comparing Alcohol Use and Congestive Heart Failure**.

Study	Study design	Country	Number of participants	% Men and women	Average age (years)	Alcohol consumption	HR/OR/RR (95% Confidence Interval)
Klatsky *et al*., 2005 [42]	Prospective Cohort	United States of America	1035	44.4% Men	74	1–2 drinks/day	RR:
				55.6% Women		3–5 drinks/day	1.0 (0.8–1.3)
						≥6 drinks/day	1.2 (0.9–1.6)
							1.7 (1.1–2.6)
Park *et al*., 2018 [43]	Prospective Cohort	South Korea	49,714	72.2% Men	49.1	15–30 g/day	OR:
				27.8% Women		30–60 g/day	1.25 (1.08–1.44)
						>60 g/day	1.33 (1.15–1.54)
							1.32 (1.11–1.57)
Larsson *et al*., 2015 [44]	Meta analysis	United States of America Canada Sweden Finland	6211	Variable among studies	21–85	14 drinks/week	RR:
							1.07 (0.77–1.48)
Whitman *et al*., 2017 [41]	Prospective Cohort	United States (California)	268,084	68.6% Men	48.8	7–14+ drinks/week	HR:
				31.4% Women			2.34 (2.29–2.39)
Sidorenkov *et al*., 2011 [45]	Cohort Autopsy Study	Russia (Arkhangelsk)	318	46.9% Male	30–70 years	7–14+ drinks/week	OR:
				53.1% Female			1.36 (0.74–2.48)
Aguilar *et al*., 2004 [46]	Prospective Cohort	Canada United States of America	2228	82.5% Men	58	1–10 drinks/week	HR:
				17.5% Women		>10 drinks/week	0.93 (0.75–1.17)
							1.25 (0.91–1.72)
Li *et al*., 2016 [47]	Prospective Cohort	China	10,824	46.1% Men	54	1–2 drinks/day	OR:
				53.9% Women		3+ drinks/day	1.183 (0.774–1.808)
							1.482 (1.117–1.965)
Yousaf *et al*., 2014 [48]	Prospective Cohort	United States (Minnesota)	2042	52.3% Men	63.1	<1 drinks/day	OR:
				47.7% Women		1–2 drinks/day	0.14 (0.04–0.43)
						>2 drinks/day	1.56 (0.39–5.20)
							4.75 (1.18–15.98)

Table notes: OR, odds ratio; HR, hazard ratio; RR, relative risk.

The toxic effects on alcohol on muscle cells are well recognized [49, 50]. In 1989, researchers demonstrated that long term alcohol use produces toxic effects on striated muscles, including heart and skeletal muscles [51]. Investigators also found that acetaldehyde, a metabolite of alcohol metabolism, negatively affected cardiac and skeletal muscle [52]. *In vitro* studies conducted on cardiomyocytes found that ethanol interferes with a number of muscle cell functions. For example, Guarnieri and Lakatta [53] demonstrated that ethanol inhibits the calcium-myofilament interaction, interfering with electromechanical coupling of muscle cell contractile filaments. Das and Harris [54] found that mitochondrial adenosine triphosphate (ATP) synthase becomes defective in the presence of alcohol leading to further loss of function in rat cardiomyocytes. Other studies found that ethanol has apoptotic effects on cardiac myocytes damaging overall function of heart [55, 56].

**Fig. 1.**
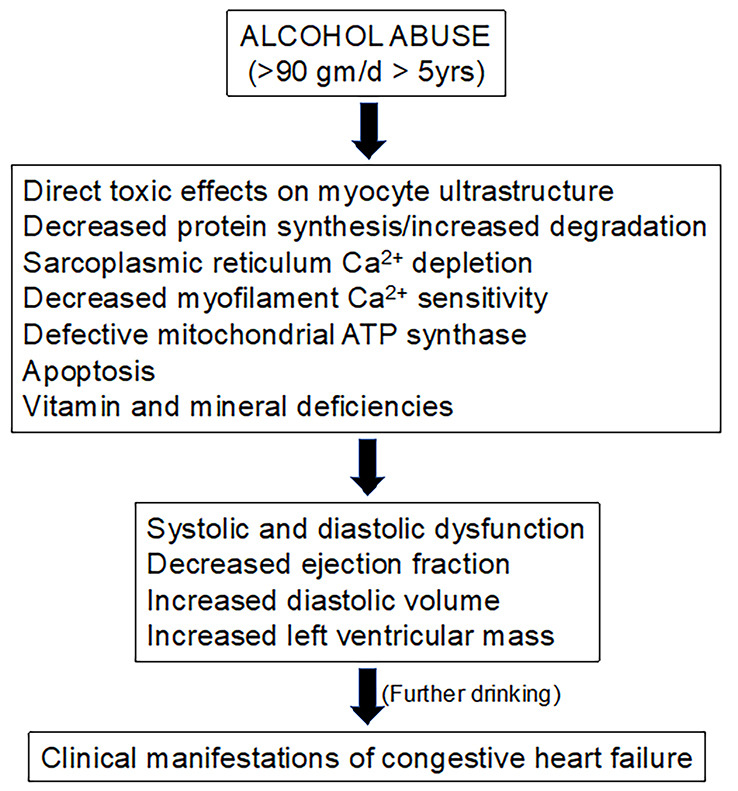
**Potential mechanisms of alcoholic cardiomyopathy.** ATP, adenosine triphosphate.

Research has shown alcohol consumption affects all areas of cell protein metabolism, from its synthesis to degradation. Human and mouse tissue studies demonstrated alcohol is a myocardial toxin and causes ultrastructural damage. Alcohol damage can include sarcoplasmic reticulum edema, contractile element fragmentation, intercalated disc expansion, and fatty deposition [57]. Alcohol produces dose dependent depression of contractile function in rat cardiomyocytes due sarcoplasmic calcium depletion [58] and decreased myofilament calcium sensitivity [59]. Studies have found an inverse relationship between use of alcohol and protein synthesis [60, 61]. Vary and colleagues [62] showed that chronic alcohol feeding in rats produced lower heart weights due to a 25% loss of cardiac proteins and a 30% reduction in the rate of protein synthesis. Potential mechanisms of alcoholic cardiomyopathy are shown in Fig. 1. Of note, Steiner and colleagues [60] reported that only chronic heavy consumption interferes with protein synthesis while moderate use has little to no effect.

Asymptomatic impairment of echocardiographic systolic and diastolic function parameters is found in most alcoholics [51]. Alcoholics have lower left ventricular ejection fraction, increased end diastolic volume, decreased mean fractional shortening and a larger mean left ventricular mass in a dose-dependent fashion [51]. Only a small percentage go on to develop overt manifestations of heart failure.

Symptomatic alcoholic cardiomyopathy is similar to other dilated cardiomyopathies. Diagnosis can be complicated by the presence of other risk factors. Taking a good history is important to rule out other risk factors, including prescribed and non-prescribed drugs (e.g., doxorubicin and cocaine), diabetes and coronary artery disease. Alcoholic cardiomyopathy is a diagnosis of exclusion.

Clinical findings of heart failure, including a S3 gallop, jugular venous dilation, cardiomegaly, and rales can be present. Co-existing alcoholic cirrhosis can lead to diagnostic confusion. Supraventricular arrhythmias (holiday heart syndrome) and sudden cardiac death are complications of alcohol abuse. Causes of death in alcoholic cardiomyopathy are similar to those with idiopathic cardiomyopathy, progressive chronic heart failure and sudden cardiac death [34]. Coexisting alcoholic cardiomyopathy and cirrhosis carries a worse prognosis [63].

Studies have not found an association between drinking in moderation and the development of cardiomyopathy. In fact, studies have reported a beneficial effect of moderate drinking in reducing mortality for patients with heart failure. For example, the Framingham heart study reported a lower incidence of heart failure with moderate alcohol consumption compared to patients who drank less than 1 drink per week [64, 65]. Another study reported a statistically significant reduction in the incidence of heart failure in individuals consuming moderate alcohol among older adults compared to those abstaining from alcohol [66]. In the Physicians Heart Study I, they observed a 56% decreased risk of heart failure in association with moderate consumption of alcohol [64].

The treatment of alcoholic cardiomyopathy is similar to that of other non-ischemic cardiomyopathies. Complete abstinence from alcohol is the mainstay of treatment, though even moderation may help. Four-year mortality for those who continue to abuse alcohol is near 50%. Treatment centers around heart failure guidelines from the European Society of Cardiology or the American College of Cardiology. Guideline-directed therapy begins with a combination of beta-blockers and an angiotensin-converting enzyme inhibitor, angiotensin receptor blocker, or angiotensin blocker-neprilysin inhibitor, and an aldosterone receptor antagonist [31]. Symptomatic management may require diuretic therapy. Co-existing nutritional deficiencies (vitamins, minerals such as magnesium, selenium, or zinc) should be supplemented. If concurrent atrial fibrillation is present, digitalis and anticoagulants may help.

Abstinence-maintaining medications, such as naltrexone, acamprosate, disulfiram, and nalmefen have shown some success in cardiomyopathy patients [67]. Spironolactone has recently been evaluated as a potential therapy in alcoholic cardiomyopathy patients, demonstrating not only therapeutic effects on treatment of cardiomyopathy, but an effect of reducing alcohol craving [68]. Little data is published for heart transplantation in alcoholic cardiomyopathy patients, and relapse would be a concern. Relapse rates in a study of liver transplants was 5.6 cases per 100 patients per year for any alcohol use and 2.5 cases per 100 patients per year for heavy alcohol use [69]. Likelihood of myocyte and contractile recovery depend first on amount and duration of alcohol consumption. Irreversible changes with cell death and fibrosis prevent recovery. For viable myocytes to recover, abstinence and compliance with guideline-directed medical therapy are necessary, as with any dilated cardiomyopathy.

## 4. Conclusions

Current guidelines and most physicians today will recommend to their patients limiting alcohol consumption to one drink per day for women, and two drinks per day for men. While epidemiological data suggests that light to moderate alcohol consumption can have advantageous cardiovascular effects, newer studies have shown that these recommendations should be tailored for each individual patient. In patients with or at higher risk for hypertension, newer studies show that even moderate drinking can lead to the progression of hypertension.

Abusing alcohol can cause a cardiomyopathy similar to other dilated cardiomyopathies. While the majority of alcoholics are clinically asymptomatic (mild systolic and diastolic dysfunction) and may never develop clinical manifestations of heart failure, a small percentage go on to develop symptomatic dilated cardiomyopathy. Because there are so many heavy drinkers, alcoholic cardiomyopathy may be the most common nonischemic dilated cardiomyopathy.

## Data Availability

Not applicable.
